# Efficacy of ultrasound-guided lumbar plexus block in reducing emergence agitation in children undergoing hip surgery

**DOI:** 10.3389/fmed.2025.1606502

**Published:** 2025-06-12

**Authors:** Xiaofeng Wang, Yonglin Chen, Hai Yan, Yongzhu Chen, Yonggang Yang, Ruyi Xing, Yu Zhang, Tao Xu, Hui Zhang

**Affiliations:** Department of Anesthesiology, Shanghai Sixth People’s Hospital Affiliated to Shanghai Jiao Tong University School of Medicine, Shanghai, China

**Keywords:** emergence agitation, general anesthesia, lumbar plexus, nerve block, pediatric, ultrasonography

## Abstract

**Objective:**

Emergence agitation (EA) is a common and challenging postoperative problem in children. We aim to investigate the effect of ultrasound-guided lumbar plexus block on emergence agitation in children undergoing hip surgery.

**Methods:**

This prospective, randomized, controlled study was conducted in children aged 1–6 year undergoing elective hip surgery. Subjects were randomly assigned to receive either ultrasound-guided lumbar plexus block combined with general anesthesia (Group Block, *n* = 172) or routine general anesthesia (Group Control, *n* = 172). The primary outcome was the incidence of EA at 30 min after emergence from general anesthesia, assessed using the Pediatric Anesthesia Emergence Delirium (PAED) scale. The secondary outcomes included the incidence of severe EA, postoperative pain evaluated by the Children’s Hospital of Eastern Ontario Pain Scale (CHEOPS) and the incidence of postoperative adverse complications.

**Results:**

The incidence of EA was significantly lower in Group Block than in Group Control (13.4% vs. 44.2%, *p* < 0.001). Group Block had a lower incidence of severe EA than Group Control (3.5% vs. 19.1%, *p* < 0.001). CHEOPS was lower in Group Block than in Group Control [mean (95%CI), 4.4(4.3–4.5) vs.4.9 (4.8–5.0), *p* < 0.001].

**Conclusion:**

Ultrasound-guided lumbar plexus block could effectively decrease the incidence and severity of emergence agitation in children undergoing hip surgery.

## Introduction

Emergence agitation (EA) in young children, especially preschool-aged children, is a common and challenging problem in the early postoperative period, characterized by a series of presentations including non-purposeful movement, inconsolability, restlessness, thrashing and agitation (1). The incidence of EA varies from 10 to 80% (2). Although EA is self-limiting and lasts for a short time about 30 min, EA causes self-injury of children, increases stress on healthcare team members (3, 4) and even leads to postoperative maladaptive behavioral changes (1, 5, 6), such as sleep disturbances, attention seeking and temper tantrums. The detailed mechanism remains unknown under this phenomenon. Consequently, it is recommended that EA should be considered as a ‘vital sign’ and has become heightened interest (1).

Various strategies have been proposed for the prevention of EA, including some pharmacological intervention and non-pharmacological strategies (1). The most favorable prevention method is not currently available (7). Many studies have found that different anesthetic techniques may influence the incidence and severity of EA (1, 8–10). Nerve block has been shown to be beneficial in emergence agitation risk reduction (11). Ultrasound-guided nerve block has become increasingly popular in pediatric orthopedic surgery in recent years, since it increases the safety and provides effective analgesia (12, 13). Moreover, pain is considered as an important factor in EA (1). Taken together, it is expected that ultrasound-guided nerve block has benefit on EA. To date, none of studies has assessed the effect of ultrasound-guided lumbar plexus block on EA.

Therefore, in this prospective, randomized and controlled study, we aimed to evaluate whether ultrasound-guided lumbar plexus block combined with general anesthesia would reduce the incidence of EA in children undergoing elective hip surgery.

## Materials and methods

### Ethics approved and registration

This prospective, single-center, randomized, controlled trial was approved by the Ethics Committee of Shanghai Sixth People’s Hospital affiliated to Shanghai Jiao Tong University School of Medicine and written informed consent was obtained from parents or guardians. The protocol was registered at the Chinese Clinical Trial Registry (Principal investigator: Hui Zhang, Registration number: ChiCTR-INR-17011525, Date of registration: 30/05/2017)^1^ and published (14). The study complied with the Declaration of Helsinki and was monitored by the Good Clinical Practice (GCP) Unit at Shanghai Sixth People’s Hospital affiliated to Shanghai Jiao Tong University School of Medicine. The study was conducted from May 2018 to February 2022 in Shanghai Sixth People’s Hospital affiliated to Shanghai Jiao Tong University School of Medicine.

### Inclusion and exclusion criteria

We screened a total of 350 children, aged 1–6 year with the ASA physical status I or II scheduled for osteotomy for developmental dislocation of the hip. Exclusion criteria included contraindication for lumbar plexus block, developmental delays, neurological or psychiatric disease, local infection at the needle entry point, coagulopathy, nerve injury, allergy to anesthetics and study medications, and lack of parental consent for the child’s participation in the study.

### Randomization and blinding

Randomization was based on computer-generated allocation and a randomization number was concealed in an opaque envelope. All participants were randomly assigned to either ultrasound-guided lumbar plexus block group (Group Block) or control group (Group Control) at a 1:1 allocation ratio after induction of general anesthesia. Anesthesiologist who performed intraoperative anesthetic care knew the assignment. Patients, parents, surgeons, assessment investigators, the medical staff who provided postoperative care in the post-anesthesia care unit (PACU), data collectors, and statisticians were all blinded to the group allocation. The nerve block details were recorded, kept in a sealed envelope and opened when medically necessary and at the beginning of statistical analysis.

### Conduct of the study

Standard monitoring including electrocardiograph (ECG), non-invasive blood pressure (NIBP), pulse oximetry (SpO2), end tidal carbon dioxide (ETCO2), and temperature were applied after all participants entered the operating room. Anesthesia induction was performed using midazolam (0.1 mg/kg), fentanyl (2 μg/kg), propofol (3 mg/kg) and vecuronium (0.1 mg/kg) administered intravenously. Patients were intubated endotracheally after successful induction, and ETCO2 was aimed at 35–40 mmHg.

Patients in Group Control received a bolus injection of fentanyl 1 μg/kg intravenously before skin incision.

**FIGURE 1:**
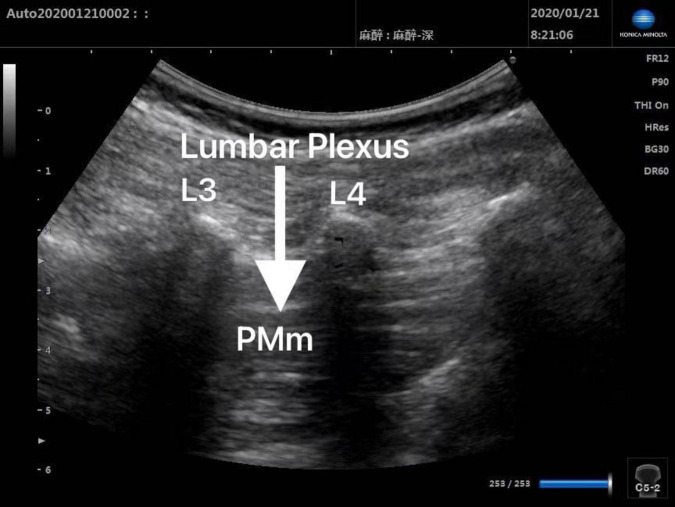
Preinjection ultrasound image of L3-4 lumbar plexus block.

**FIGURE 2:**
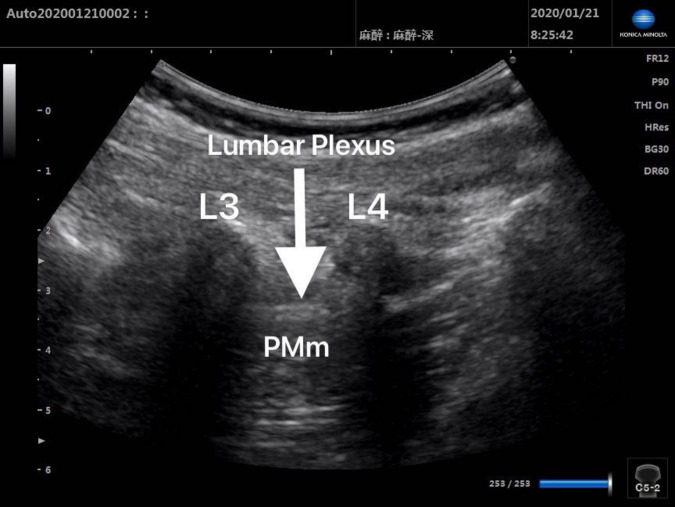
Postinjection ultrasound image of L3-4 lumbar plexus block.

Patients in Group Block received ultrasound-guided lumbar plexus block before skin incision. Ultrasound scans of lumbar plexus were carried out using the S-NerveTM Ultrasound System (Sonosite Inc., Bothell, WA, United States) in patients assigned to Group Block. A linear array (6∼13 MHz) transducer was used to perform lumbar plexus block with the longitudinal approach. All nerve blocks in this study were performed by the same experienced anesthesiologist, who was qualified for ultrasound-guided lumbar plexus block. After induction, every patient assigned to Group Block was placed in the lateral decubitus position with the operative side upwards and the hips and knees flexed in order to enlarge interspinous spaces and provide better image of lumbar plexus. When transducer was placed 1.5∼2 cm lateral to the midline of the patient’s back and parallel to the long axis of the spine at the level L3-4, the lumbar transverse processes produced a “trident sign” (15). Because the psoas major muscle is anterior to the transverse processes, the lumbar plexus was distinguished within the major psoas muscle (Figures 1, 2). With ultrasound guidance, the block needle was advanced cautiously perpendicular to the skin until the needle tip was located 1–1.5 cm below the space between the transverse processes. All patients received 0.2% ropivacaine 1 ml/kg (Naropin 10 mg/ml, Astrazeneca, Wilmington, DE, United States) (16). The maximum dose of ropivacaine was limited to 20 ml (16).

The success of lumbar plexus block was defined as follows: (i) visualization of the needle tip in the right position, the spread of local anesthetic around the lumbar plexus nerve; (ii) an increase of no more than 15% in heart rate, blood pressure in response to the skin incision; (iii) an increase of no more than 25% in heart rate, blood pressure during the operation (17). Patients in Group Block were considered as failed blocks once one of the above criteria could not be met. The surgery was performed at least 15 min after the nerve block.

Anesthesia was maintained with 60% nitrous oxide, 40% oxygen and 2% end-tidal sevoflurane. The concentration of sevoflurane was added by 0.5% if the values of heart rate, blood pressure increased more than 15% from the baseline values during the operation. Conversely, the concentration of sevoflurane was reduced by 0.5% if the values of heart rate, blood pressure decreased more than 15% from the baseline values. If the increase was more than 25% from the baseline values, additional bolus of fentanyl 1 μg/kg was administered. Patients recorded as a failure or an inadequate lumbar plexus block were followed up. Intention-to-treat analysis was used to analyze data including failed block.

### Intraoperative anesthetic management and postoperative assessments

During the operation, if the heart rate reduced to less than 50 beats per min, atropine 10 μg/kg was used. Ephedrine 0.1 mg/kg was used to treat hypotension when blood pressure decreased more than 25% from the baseline value. Intraoperative data collection included heart rate, blood pressure, respiratory rate, concentration of sevoflurane, and fentanyl dose. The average value of end-tidal sevoflurane concentration (EtSev%) was calculated from all values recorded every 5 min throughout the operation.

At the end of the operation, sevoflurane and nitrous oxide were discontinued and oxygen flow was increased to 6 L/min. Patients were transferred to PACU and monitored until the end of the study. After completion of the operation, when patients remained hemodynamic stability, regained adequate spontaneous respiratory rate (>20/min), full eye opening or purposeful movement, extubation was performed in PACU. After extubation and emergence, a blinded well-trained observer performed all assessments. EA was assessed by PAED scale (18) at 0, 5, 10, 20, and 30 min after emergence. We defined EA as PAED scale ≥ 10 (18, 19). Severe EA was defined as PAED score ≥ 13 (18) and treated with a bolus of fentanyl 0.5 μg/kg (8, 18). The primary outcome was the incidence of EA 30 min after emergence from anesthesia. Postoperative pain at 0, 5, 10, 20, and 30 min after emergence was evaluated by the CHEOPS scale (20). Patient with CHEOPS > 7 was treated with a bolus of fentanyl 0.5 μg/kg (8, 18).

Emergence time (from discontinuation of sevoflurane to the first response to a simple verbal command), extubation time (from the end of anesthesia to extubation), intraoperative collective fentanyl rescue dose, adverse effects and complications were recorded.

At the end of study, that is, 30 min after emergence, the patients were given nurse controlled analgesia (NCA) with morphine. Bolus dose is 10 μg/kg, lockout interval 30 min and background infusion is 10 μg/kg/h.

## Statistical analysis

The incidence of EA for pediatric hip surgery was approximately 42% in our pilot study. Therefore, a minimum sample size of 172 patients in each group would have 80% power to detect an absolute reduction of 15% in the incidence of EA in the intervention group at a significance level of 0.05, considering a possible dropout rate of 10%.

Categorical variables such as the incidence of EA and severe EA were expressed as frequencies (percentage) and analyzed with Chi-square test or Fisher’s exact test. One-way Repeated-measures ANOVA or Generalized estimated equation (GEE) was applied to analyze data for PAED and CHEOPS according to the test for normality and homogeneity of variance. Continuous variables with a normal distribution were summarized with mean (standard deviation, SD) and analyzed with an independent-sample *t*-test.

The level of significance for each test was set at α = 0.05, 2 tailed. All data were subjected to the Kolmogorov-Smirnov test for normality. All data were analyzed with SPSS 23.0 (IBM SPSS Statistics for Windows, Version 23.0; IBM Corp., Armonk, NY). Intention-to-treat strategy was performed in every case.

## Results

**FIGURE 3:**
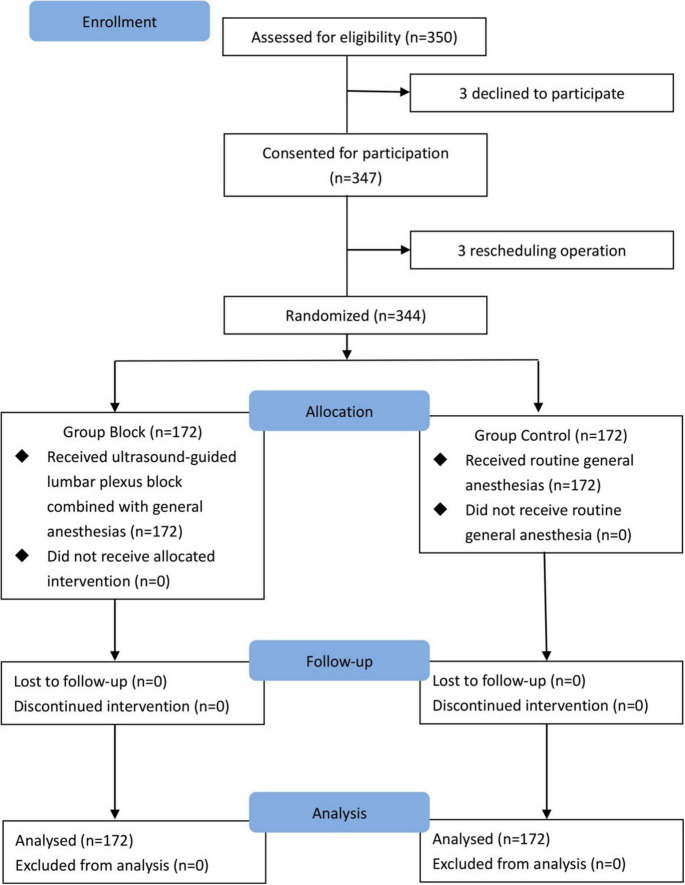
CONSORT flowchart.

A total of 344 subjects were randomly assigned to two groups (*n* = 172 per group) and completed the study (Figure 3). All children in Group Block received an effective lumbar-plexus block, as evidenced by the fact that all criteria for a successful block were met in each case. Table 1 shows patients baseline demographics and clinical characteristics. There were no significant differences between groups.

**TABLE 1:** Patients baseline demographics and clinical characteristics.

Demographics characteristics	Group block (*n* = 172)	Group control (*n* = 172)	*P*-value
Age (years)	4.2 (0.93)	4.1 (0.90)	0.98
Weight (kg)	16.6 (1.9)	16.5 (1.7)	0.67
Sex (male/female)	80/92	82/90	0.60
ASA classification (I/II)	172/0	172/0	NA
mYPAS	8.1 (1.6)	7.9 (1.1)	0.18
Duration of surgery (min)	120.3 (6.5)	119.1 (5.9)	0.52
Duration of anesthesia (min)	141.6 (5.1)	142.4 (4.8)	0.45

Data are presented as mean (standard deviation) or number of patients. mYPAS, modified Yale Preoperative Anxiety Scale; ASA, American Society of Anesthesiologists.

**FIGURE 4:**
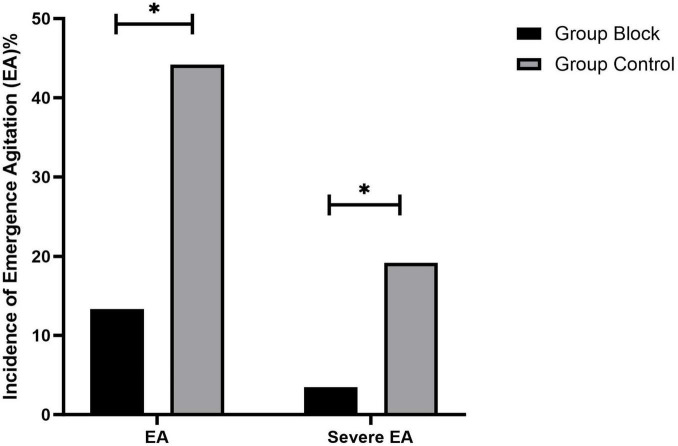
Incidence of emergence agitation (EA). *P < 0.01 vs. group control.

For the primary outcome, there was a significant difference between the two groups in the incidence of EA 30 min after emergence from anesthesia, with a lower incidence in Group Block than in Group Control [13.4% (23/172)vs.44.2% (76/172), odds ratio (OR) 0.195, 95% confidence interval (CI) 0.115 to 0.332, *p* < 0.001, Figure 4]. Similarly, the percentage of patients with severe EA was significantly lower in Group Block than in Group Control [3.5% (6/172) vs. 19.1% (33/172), odds ratio (OR) 0.152, 95% confidence interval (CI) 0.062 to 0.374, *p* < 0.001, Figure 4].

A generalized estimated equation was applied to analyze data for PAED, showing that lumbar-plexus block was associated with a decrease in PAED scores (Wald *X*^2^ = 19.5, *p* < 0.001, Table 2). Subjects in both groups had the highest PAED scores at emergence and trended down to below 5 at 10 min after emergence. CHEOPS was analyzed by a generalized estimated equation, showing a significant difference between groups (Wald *X*^2^ = 50.2, *p* < 0.001, Table 2).

**TABLE 2:** Patients outcomes in PACU.

Variables	Group block (*n* = 172)	Group control (*n* = 172)	*P*-value
Mean PAED (mean, 95%CI)	5.6 (5.3–6.0)	6.9 (6.6–7.2)	<0.001
Mean CHEOPS (mean, 95%CI)	4.4 (4.3–4.5)	4.9 (4.8–5.0)	<0.001
Post-anesthesia complications			
PONV (n)	1	2	0.559
Desaturation (n)	1	3	0.309
Laryngospasm (n)	0	0	NA
Emergence time (min) (mean, SD)	10.7 (2.0)	18.5 (2.4)	<0.001
Extubation time (min) (mean, SD)	15.9 (2.2)	23.9 (2.3)	<0.001

NA, not applicable; PONV, postoperative nausea or vomiting.

**FIGURE 5:**
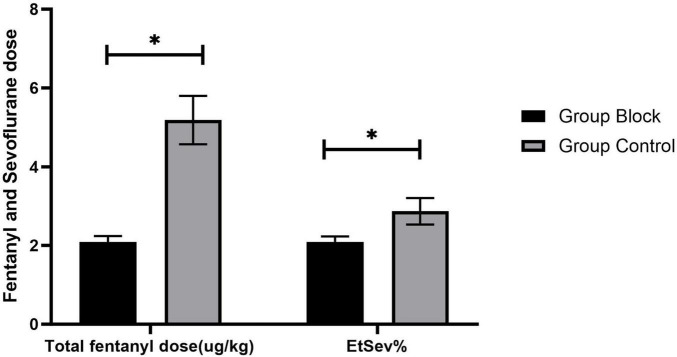
Fentanyl and sevoflurane dose. *P < 0.01 vs. group control.

The average value of end-tidal sevoflurane (EtSev%) in Group Block was significantly less than in Group Control [2.09 (0.14) vs. 2.87 (0.34), *p* < 0.001, Figure 5]. In summarize, total fentanyl dose (μg/kg) was significantly less in Group Block than in Group Control [2.09(0.15) vs. 5.19 (0.61), *p* < 0.001, Figure 5]. Significantly fewer patients in Group Block need fentanyl rescue in PACU compared to Group Control [10.5% vs. 34.9%, odds ratio (OR) 0.218, 95% confidence interval (CI) 0.122 to 0.390, *p* < 0.001].

There was no case treated with atropine or ephedrine. In Group Control, two cases of blood pressure decreased more than 15% and less than 25% from the baseline values. The concentration of sevoflurane was reduced by 0.5%. No significant difference was observed in the incidence of postoperative adverse events between the two groups (Table 2). There was no case of local anesthetic intoxation and nerve damage in Group Block. Emergence time and extubation time were significantly shorter in Group Block than in Group Control (Table 2).

## Discussion

This prospective, randomized and controlled study revealed that ultrasound-guided lumbar plexus block could decrease the incidence and severity of EA effectively and offer better analgesia in children aged 1–6 year undergoing elective hip surgery. Furthermore, ultrasound-guided lumbar plexus block reduced the use of fentanyl and sevoflurane.

The precise etiology of EA remains unclear (1). Amongst numerous factors associated with EA, pain plays an important role in EA. Adequate analgesia has been shown to mitigate the incidence of EA. Several studies have highlighted that nerve block offer effective analgesia (12, 13, 15, 21). However, the impact of nerve blocks on EA remains inconclusive due to inconsistent findings in prior research (1). Some studies have demonstrated that nerve block can reduce the incidence of EA (8, 9). The primary rationale for this benefit is the provision of satisfactory postoperative analgesia and a notable reduction in sevoflurane usage (8). Conversely, Ohashi et al. (17) found ilioinguinal/iliohypogastric block did not influence the incidence of EA. They attributed this outcome to the minimally invasive nature of the surgeries studied, suggesting that such blocks might be more beneficial in more invasive procedures. In this study, we focused on osteotomy in children with developmental dislocation of the hip, a common pediatric procedure associated with significant postoperative pain. Previous studies on the effect of nerve blocks on EA were predominantly limited to short-term procedures with minimal to moderate pain and involved several superficial nerves (8, 9, 17). Our research aims to evaluate the effect of lumbar plexus block, a deeper and more complex procedure, on EA following long-term surgery. The advent of ultrasound technology has transformed pediatric anesthesia, with ultrasound-guided nerve blocks gaining popularity due to their efficacy (16, 22). Our findings indicated that the application of ultrasound-guided lumbar plexus block significantly reduce both the incidence and severity of EA in children undergoing hip surgery. Additionally, Group Block exhibited lower pain scores and reduced total fentanyl consumption compared to Group Control. Consistent with previous reports, our results suggest that nerve blocks can decrease the incidence of EA by providing effective analgesia (8, 9). This alignment with existing literature underscores the potential benefits of lumbar plexus blocks in managing EA in pediatric patients undergoing painful, long-duration surgeries. Thus, our study contributes to the growing body of evidence supporting the use of advanced nerve block techniques in pediatric anesthesia to improve postoperative outcomes. The number of patients requiring fentanyl rescue was significantly lower in Group Block compared to Group Control. We hypothesize that the superior analgesic efficacy of the ultrasound-guided lumbar plexus block played a pivotal role in reducing the incidence of EA. This assumption aligns with the well-established principle that effective pain management can mitigate EA, as inadequate analgesia is a known contributing factor. Furthermore, the shorter emergence and extubation times observed in Group Block compared to Group Control may be attributed to the reduced fentanyl consumption in the former group. This reduction in opioid use is likely a direct consequence of the effective analgesia provided by the lumbar plexus block, which not only alleviates pain but also minimizes the need for systemic opioids, thereby facilitating faster recovery and smoother emergence from anesthesia. These findings collectively support the hypothesis that ultrasound-guided lumbar plexus block, by enhancing analgesia and reducing opioid requirements, contributes to a lower incidence of EA and improved recovery profiles in pediatric patients undergoing hip surgery.

Distinguishing between pain and EA is challenging (1). In patients who are likely to experience postoperative pain, pain may act as a confounding factor in studies of EA. To diagnose EA in children, we utilized the Pediatric Agitation and Emergence Delirium (PAED) scale, which is currently the standard tool for this purpose (11). Given that fentanyl is considered a first-line agent in managing agitation (11), we used fentanyl as the rescue medication for agitation (8, 18). To minimize the confounding effect of pain on EA, we administered sufficient fentanyl to ensure adequate analgesia in both groups. Patients with a Children’s Hospital of Eastern Ontario Pain Scale (CHEOPS) score of > 6 were considered to have pain (23). Our results showed that the mean CHEOPS scores in both groups were lower than 6, indicating that the patients received effective pain relief. Therefore, the patients experiencing EA were indeed exhibiting agitation rather than pain.

The PAED scale was validated to reflect the presence of EA. Generalized estimated equation analysis for PAED showed the lumbar plexus block was significantly associated with a reduction in PAED scale, suggesting its potential role in mitigating EA. Our study demonstrated a downward trend in PAED scores following emergence, which aligns with the findings of Frederick et al. (24). Specifically, PAED scale peaked at the time of emergence and gradually declined to below 5 within 30 min post-emergence in both groups. This pattern confirms that EA is typically self-limiting, with a duration of approximately 30 min. These results not only reinforce the transient nature of EA but also highlight the potential benefits of lumbar plexus block in reducing both the intensity and duration of EA, thereby improving recovery outcomes in pediatric patients.

The use of sevoflurane has been associated with a high incidence of EA (11). Data from clinical studies suggest that intrinsic effect of sevoflurane is associated with EA (25–27) and dose reduction of sevoflurane might be a major contributor for reducing the incidence of EA (19). As a consequence, reducing the use of sevoflurane is strongly considered in children with a high risk of EA (11). The average value of end-tidal sevoflurane concentration in Group Block was significantly lower than in Group Controlin our study. Ultrasound-guided lumbar plexus block reduced the amount of sevoflurane. Thus, the decreased amount of sevoflurane could be another mechanism by which lumbar plexus block reduces the incidence of EA. Recent studies (28, 29) have reported that nerve blockage might attenuate postoperative inflammation to improve postoperative cognitive impairment. This could also be a potential mechanism of effect of lumbar plexus block on EA in our study, which needs to be defined in further studies.

Whatever the mechanism of lumbar plexus block on EA, lumbar plexus block provides better control of pain and reduction of sevoflurane use and hence ameliorates EA.

Although benefits of ultrasound-guided lumbar plexus block on EA were achieved by this prospective, randomized study, there are several limitations in our study. Our study was limited to 30 min after emergence because EA lasts for a short time about 30 min mostly. Further study is required to elucidate if lumbar plexus block could improve long-term effect especially maladaptive behavior (1). The second limitation is that the target lumbar plexus would be more precise if ultrasound guidance was combined with nerve stimulation. However, this limitation may be offset somewhat because the ultrasound landmarks of lumbar plexus in children are more easily identified when compared with adults. To this end, we drew up a standard procedure of nerve block and could ensure satisfactory regional block in our study.

## Conclusion

In conclusion, our findings indicate that the incidence and severity of EA in children undergoing hip surgery are decreased when applying ultrasound-guided lumbar plexus block. Lumbar plexus block appears to be a promising and practical option for mitigating EA in children following general anesthesia.

## Data Availability

The original contributions presented in this study are included in this article/supplementary material, further inquiries can be directed to the corresponding authors.

## Footnotes

1
http://www.chictr.org.cn/
